# NACA and LRP6 Are Part of a Common Genetic Pathway Necessary for Full Anabolic Response to Intermittent PTH

**DOI:** 10.3390/ijms23020940

**Published:** 2022-01-15

**Authors:** René St-Arnaud, Martin Pellicelli, Mahmoud Ismail, Alice Arabian, Toghrul Jafarov, Chengji J. Zhou

**Affiliations:** 1 Research Centre, Shriners Hospital for Children—Canada, Montreal, QC H4A 0A9, Canada; martin.pellicelli@gmail.com (M.P.); MIsmail@shriners.mcgill.ca (M.I.); aarabian@shriners.mcgill.ca (A.A.); jafarovta@yahoo.com (T.J.); 2 Department of Surgery, Faculty of Medicine and Health Sciences, McGill University, Montreal, QC H3G 1A4, Canada; 3 Department of Human Genetics, Faculty of Medicine and Health Sciences, McGill University, Montreal, QC H3A 0C7, Canada; 4 Department of Medicine, Faculty of Medicine and Health Sciences, McGill University, Montreal, QC H3A 1A1, Canada; 5 Department of Biochemistry and Molecular Medicine, School of Medicine, University of California at Davis, Sacramento, CA 95817, USA; cjzhou@ucdavis.edu; 6 Institute for Pediatric Regenerative Medicine, Shriners Hospitals for Children—Northern California, Sacramento, CA 95817, USA

**Keywords:** NACA, LRP6, parathyroid hormone, osteoblasts, osteocytes, bone

## Abstract

PTH induces phosphorylation of the transcriptional coregulator NACA on serine 99 through Gαs and PKA. This leads to nuclear translocation of NACA and expression of the target gene *Lrp6*, encoding a coreceptor of the PTH receptor (PTH1R) necessary for full anabolic response to intermittent PTH (iPTH) treatment. We hypothesized that maintaining enough functional PTH1R/LRP6 coreceptor complexes at the plasma membrane through NACA-dependent *Lrp6* transcription is important to ensure maximal response to iPTH. To test this model, we generated compound heterozygous mice in which one allele each of *Naca* and *Lrp6* is inactivated in osteoblasts and osteocytes, using a knock-in strain with a *Naca*^99^ Ser-to-Ala mutation and an *Lrp6* floxed strain (test genotype: *Naca*^99S/A^; *Lrp6*^+/fl^;OCN-Cre). Four-month-old females were injected with vehicle or 100 μg/kg PTH(1-34) once daily, 5 days a week for 4 weeks. Control mice showed significant increases in vertebral trabecular bone mass and biomechanical properties that were abolished in compound heterozygotes. *Lrp6* expression was reduced in compound heterozygotes vs. controls. The iPTH treatment increased *Alpl* and *Col1a1* mRNA levels in the control but not in the test group. These results confirm that NACA and LRP6 form part of a common genetic pathway that is necessary for the full anabolic effect of iPTH.

## 1. Introduction

Administering PTH at a low dosage once a day (intermittent PTH; iPTH) promotes bone formation through pleiotropic effects on osteoblasts and osteocytes [1,2,3]. Results from numerous studies show that multiple signaling pathways act in parallel or synergistically to achieve the full anabolic response to iPTH treatment (reviewed in [4,5]).

We characterized a PTH-initiated signaling cascade that involves the transcriptional coregulator NACA (alpha chain of the nascent polypeptide-associated complex; αNAC) [6]. Upon PTH binding to its receptor (PTH1R), Gα_s_ is activated to stimulate cAMP accumulation and PKA activity, resulting in phosphorylation of NACA, a protein shuttling between the cytoplasm and the nucleus. Phosphorylation of NACA on residue Serine 99 (99S) by PKA sends it to the nucleus [6], where it assembles with transcription factors such as JUN or JUND homodimers and components of the general transcriptional machinery to activate transcription of the osteocalcin (*Bglap2*) and *Lrp6* (*Low density lipoprotein receptor-Related Protein* 6) genes [7,8,9,10]. This function of NACA as a transcriptional coregulator of osteogenic target genes is physiologically relevant to control bone mass [6,8].

LRP6 (and its related family member LRP5) activates canonical Wnt signaling by complexing with Wnt ligand-bound Frizzled receptors [11,12], which in bones leads to increased formation [13]. This role of LRP6 was confirmed in several mutant mouse models [14,15] as well as in high bone mass patients with gain-of-function missense mutations in LRP6 [16,17].

LRP6 also acts as a coreceptor with PTH1R to allow optimal activation of the PTH signaling pathway in osteoblasts and osteocytes. When *Lrp6* expression is disrupted in mice, the anabolic action of iPTH is blunted [18]. The amount of LRP6 available to complex with PTH1R at the plasma membrane affects PTH responses. For example, N-Cadherin (encoded by the *Cdh2* gene) sequesters LRP6 away from the cell surface [19], and ablation of *Cdh2* in mice increases membrane PTH1R/LRP6 complexes. This in turn promotes signaling downstream from PHTR1, enhancing bone formation following iPTH treatment [20,21]. We hypothesized that NACA plays a role in achieving maximal iPTH responses by controlling *Lrp6* transcription to maintain an optimal amount of PTH1R/LRP6 complex at the plasma membrane.

We tested this hypothesis using site-directed mutagenesis of NACA at residue 99S in a knock-in mice model and through gene dosage manipulation in compound heterozygous mice in which one allele each of *Naca* and *Lrp6* is inactivated in osteoblasts and osteocytes. Our results show that the PTH-PKA-NACA pathway is necessary for the full anabolic effect of iPTH and that *Naca* and *Lrp6* are part of a common genetic pathway regulating bone mass downstream of iPTH treatment.

## 2. Results

### 2.1. Phenotype of Naca^99S/A^ Mice

A serine-to-alanine (99S/A) mutation of NACA 99S introduces a non-phosphorylatable residue that attenuates nuclear entry resulting in transcriptional inactivity [6]. We characterized the phenotype of a knock-in mouse strain in which serine residue 99 was replaced by an alanine to generate the *Naca*^99S/A^ strain. All genotypes grew normally and there was no difference between the weights of homozygous wild-type (*Naca*^99S/S^), heterozygous (*Naca*^99S/A^), or homozygous mutant (*Naca*^99A/A^) male or female littermates at 6 weeks of age (Supplementary Figure S1A,B). Female but not male mutant mice were heavier than littermates at 12 weeks (Supplementary Figure S1C,D).

We analyzed vertebrae from all genotypes by microcomputed tomography (μCT) and observed very minor differences in trabecular and cortical bone parameters that did not impact the biomechanical properties of the vertebrae from *Naca*^99A/A^ mutant mice. At 6 weeks of age, male mutant mice had slightly reduced bone mass (BV/TV, Supplementary Figure S2C), but this was transient and not observed at 12 weeks of age (Supplementary Figure S2G). All other trabecular parameters did not show significant differences at either 6 or 12 weeks of age (Supplementary Figure S2). The only cortical parameter showing a significant difference was thickness in 6-week-old males (Supplementary Figure S3D), but this was also transient and did not recur at 12 weeks of age (Supplementary Figure S3). As previously mentioned, these subtle changes had no impact on the biomechanical properties of the vertebrae in either sex, at any age (Supplementary Figure S4). We conclude that inactivating the PKA phosphoacceptor site of NACA has no impact on steady-state bone homeostasis.

**Figure 1:**
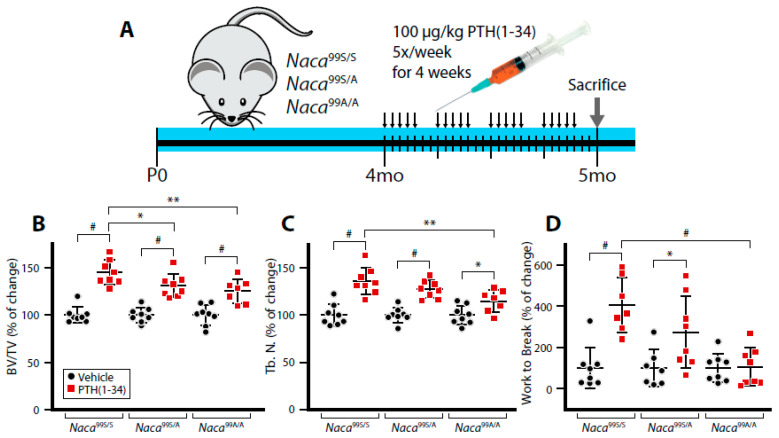
Mutation of the PKA phosphoacceptor site within the NACA sequence impairs the osteoanabolic response to intermittent PTH treatment. Four-month-old female mice were treated once daily, 5 days/week for 1 month with 100 μg/kg of PTH(1-34) (**A**). Vertebrae were analyzed by μCT (**B**,**C**) and compression testing (**D**). BV/TV, bone volume/tissue volume; Tb. N., trabecular number. Results are expressed as change from vehicle-treated animals, which are ascribed a value of 100%. * *p* < 0.05; ** *p* < 0.01; ^#^ *p* < 0.0001, two-way analysis of variance (ANOVA) with post hoc tests.

We then treated 4-month-old females with vehicle or 100 μg/kg of PTH(1-34), 5 times per week for 4 weeks prior to sacrifice at 5 months of age (Figure 1A). In response to intermittent injection of PTH, the increase in vertebral trabecular bone volume (Figure 1B) and trabecular number (Figure 1C) was significantly blunted in heterozygous (*Naca*^99S/A^) and homozygous mutant (*Naca*^99A/A^) littermates. Moreover, the increase in biomechanical resistance of iPTH-treated vertebrae was lost in homozygous mutant animals (Figure 1D). These results show that phosphorylation of residue 99S is essential to mediate the full response to iPTH treatment and confirm the physiological role played by NACA in the bone-anabolic function of PTH.

### 2.2. Phenotype of Compound Naca^99S/A^;Lrp6^+/fl^;Ocn-Cre Heterozygous Mice (Compound Heterozygotes)

To determine if *Naca* and *Lrp6* form part of a common genetic cascade, we generated cohorts of compound heterozygous mice in which one allele each of *Naca*^99S^ and *Lrp6* was inactivated in osteoblasts and osteocytes using the osteocalcin (OCN)-Cre driver [22]. Our initial breeding strategy generated the following genotypes in equivalent proportion: *Naca*^99S/S^;*Lrp6*^+/fl^ (wild-type), *Naca*^99S/S^;*Lrp6*^+/fl^;OCN-Cre (one allele of *Lrp6* inactivated), *Naca*^99S/A^;*Lrp6*^+/fl^ (one allele of Naca inactivated) and *Naca*^99S/A^;*Lrp6*^+/fl^;OCN-Cre (one allele of each gene inactivated, compound heterozygotes). There were no gross phenotypic manifestations for any of the genotypes studied (data not shown).

**Figure 2:**
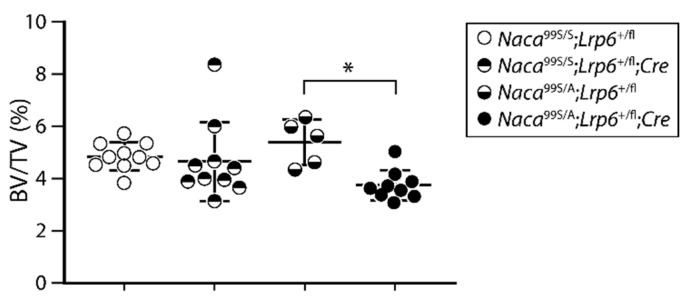
*Naca* and *Lrp6* form part of a common genetic pathway regulating bone mass. Femurs of female mice were analyzed by μCT at 3 months of age. BV/TV, bone volume/tissue volume. * *p* < 0.05, two-way ANOVA with post hoc tests.

Mice heterozygous for either *Lrp6* (*Naca*^99S/S^;*Lrp6*^+/fl^;OCN-Cre) or *Naca* (*Naca*^99S/A^;*Lrp6*^+/fl^) alone exhibited no differences in femoral bone volume compared to wild-type (*Naca*^99S/S^;*Lrp6*^+/fl^) littermates (Figure 2). The combined loss of one *Lrp6* allele and one *Naca* allele in bone cells caused a significant decrease in bone volume in compound *Naca*^99S/A^; *Lrp6*^+/fl^;OCN-Cre mice compared to control genotypes (Figure 2). These results show that *Naca* and *Lrp6* are part of a common genetic pathway impacting bone mass homeostasis.

### 2.3. Response of Compound Heterozygotes to iPTH

**Figure 3:**
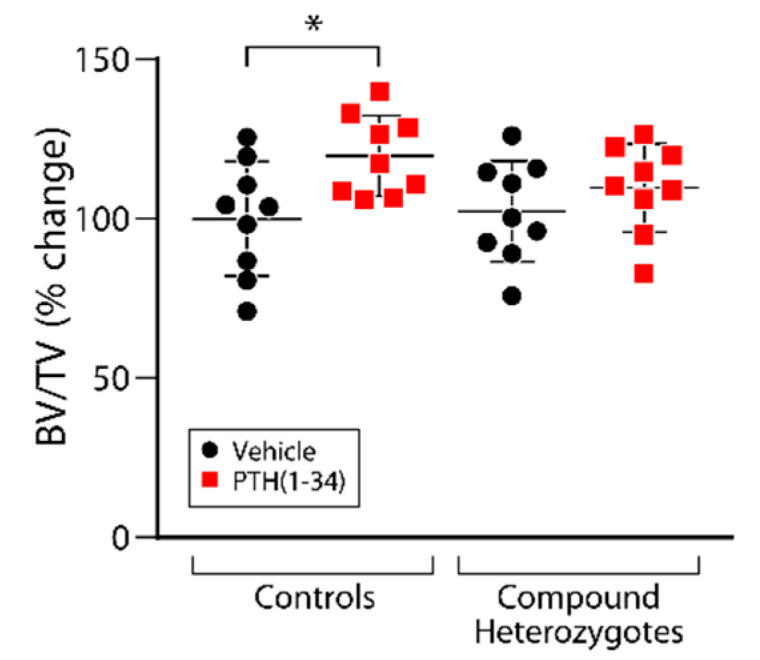
The osteoanabolic response to iPTH is inhibited in *Naca*/*Lrp6* compound heterozygotes. Four-month-old female mice were treated once daily, 5 days/week for 1 month with 100 μg/kg of PTH(1-34). Vertebrae were analyzed by μCT. BV/TV, bone volume/tissue volume. Results are expressed as change from vehicle-treated animals, which are ascribed a value of 100%. * *p* < 0.05, two-way ANOVA with post hoc tests.

**Figure 4:**
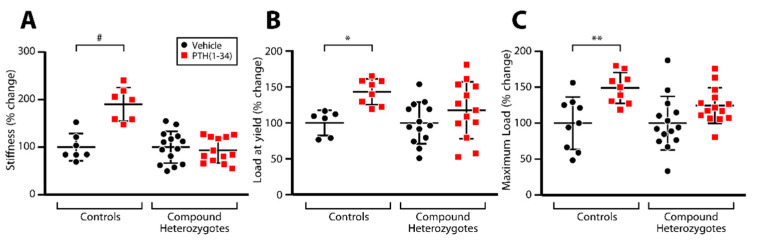
The increase in biomechanical properties induced by iPTH treatment is blunted in *Naca*/*Lrp6* compound heterozygotes. Four-month-old female mice were treated once daily, 5 days/week for 1 month with 100 μg/kg of PTH(1-34). L2 vertebrae were analyzed by compression testing to measure stiffness (**A**), load at yield (**B**) and maximum load (**C**). Results are expressed as change from vehicle-treated animals, which are ascribed a value of 100%. * *p* < 0.05; ** *p* < 0.01; ^#^ *p* < 0.0001, two-way analysis of variance (ANOVA) with post hoc tests.

We next assessed the response of compound heterozygous mice to iPTH treatment. Since the control genotypes tested exhibited comparable osteoanabolic responses to iPTH (Supplementary Figure S5), we used littermates of the *Naca*^99S/S^; *Lrp6*^+/+^; OCN-Cre genotype as controls (to rule out any effect of the Cre transgene) to compare with the *Naca*^99S/A^; *Lrp6*^+/fl^; OCN-Cre test genotype (compound heterozygotes). Inactivating one allele each of *Naca* and *Lrp6* obliterated the increase in vertebral trabecular bone mass (Figure 3) and biomechanical properties (Figure 4) induced by iPTH treatment in control mice.

**Figure 5:**
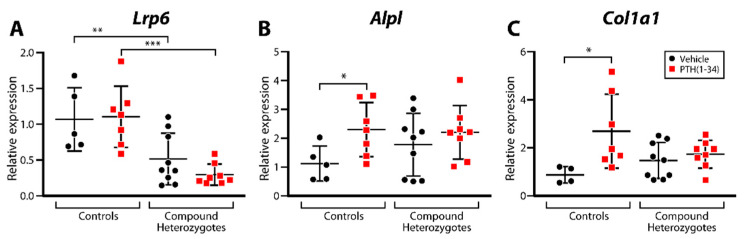
Gene expression monitoring in control (*Naca*^99S/S^;*Lrp6*^+/+^;OCN-Cre) and compound heterozygous (*Naca*^99S/A^;*Lrp6*^+/fl^;OCN-Cre) littermates. RNA was extracted from marrow-flushed tibial diaphyses, reverse-transcribed and analyzed by RT-qPCR. Relative expression of *Lrp6* (**A**), *Alpl* (**B**) and *Col1a1* (**C**) is shown. * *p* < 0.05; ** *p* < 0.01; *** *p* < 0.001, two-way ANOVA with post hoc tests.

The expression of *Lrp6* was reduced in compound heterozygous animals (Figure 5A), although we did not detect changes in *Naca* expression (data not shown). In response to iPTH treatment, control mice showed increased expression of alkaline phosphatase (*Alpl*, Figure 5B) and the alpha 1 chain of type I collagen (*Col1a1*, Figure 5C), but this was not observed in compound heterozygotes (Figure 5).

**Figure 6:**
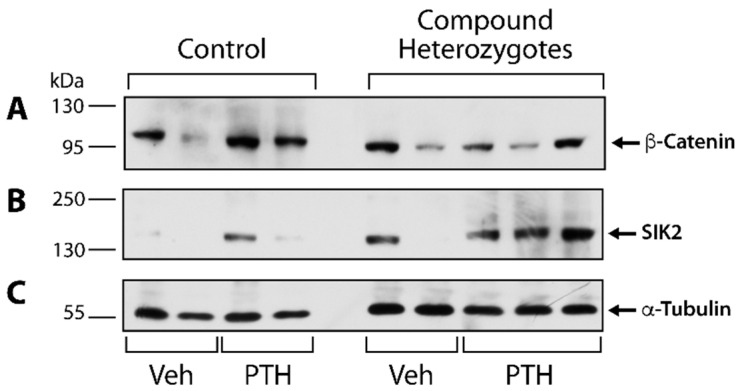
Parallel PTH signaling pathways in control (*Naca*^99S/S^;*Lrp6*^+/+^;OCN-Cre) and compound heterozygous (*Naca*^99S/A^;*Lrp6*^+/fl^;OCN-Cre) littermates. Protein extracts were prepared from tibial shafts and immunoprobed for β-Catenin (**A**) and SIK2 (**B**). Equivalent loading was assessed by probing for α-Tubulin (**C**).

We assessed the impact of gene dosage manipulation for *Naca* and *Lrp6* on other signaling pathways acting downstream from iPTH administration. We observed sample-to-sample variation in low-abundance protein recovery from TRIzol extracts (Figure 6A,B). Despite this fact, we can assess that total β-catenin was increased by iPTH treatment in control littermates but not noticeably in compound heterozygotes (Figure 6A). On the contrary, there were no major changes in SIK2 protein levels following iPTH injections in controls, but SIK2 was markedly enhanced in compound heterozygotes after the iPTH regimen (Figure 6B), suggesting potential compensatory mechanisms.

## 3. Discussion

*Lrp6* was identified as a candidate NACA target gene following Chromatin Immunoprecipitation with deep sequencing (ChIP-Seq) against NACA in MC3T3-E1 osteoblastic cells treated with vehicle or PTH(1-34) [10]. RNA-Seq was performed in parallel. We confirmed that *Lrp6* showed increased NACA binding to its proximal promoter and increased expression following PTH treatment, establishing it as a bona fide NACA transcriptional target. These experiments using an established osteoblastic cell line allowed to understand the transcriptional control of the expression of *Lrp6* downstream from PTH stimulation [10], but the physiological relevance of our findings remained to be established. In the current study, we used gene dosage manipulation to confirm the genetic interaction between *Naca* and *Lrp6*, and to establish their importance for the osteoanabolic response to iPTH in vivo.

We first generated the relevant *Naca* mutant strain by mutating the PKA phosphoacceptor residue serine 99 into an alanine, a mutation that prevents nuclear translocation of NACA and inhibits its transcriptional coactivation function [10]. Steady-state bone homeostasis was not affected in *Naca*^99A/A^ mutants (Supplementary Figures S2–S4), suggesting that the PKA-NACA pathway is redundant in the absence of specific stimuli. However, *Naca*^99A/A^ mutant mice exhibited a blunted response to iPTH treatment, demonstrating that the PTH-PKA-NACA signaling cascade is essential for maximal iPTH bone anabolic stimulation.

Our results suggest that the transcriptional control of *Lrp6* expression is one of the mechanisms through which the PKA-NACA pathway promotes maximal iPTH-dependent bone mass gain. Even in the absence of cyclic PTH administration, bone homeostasis was affected by inactivating one allele each of *Naca* and *Lrp6*, demonstrating that both genes form part of a common genetic pathway. Under these steady-state conditions, however, it is possible that a reduction in surface LRP6 expression may blunt WNT signaling and contribute to the reduced bone volume observed [14].

The impact of modifying gene dosage for *Naca* and *Lrp6* on the response to iPTH treatment was dramatic, as gains in bone mass and increases in biomechanical properties of treated bones were eliminated in compound heterozygotes.

Considering the numerous pathways acting downstream from PTH stimulation to reach maximal anabolic response to iPTH treatment [4,5], we anticipated that the genetic manipulation we generated would result in a blunted response and not a full inhibition. The data suggest that under the experimental conditions used, there is no redundancy of function from other pathways that can compensate for the loss of NACA-dependent *Lrp6* expression downstream from PTH.

The WNT/LRP6/β-catenin axis is involved in the anabolic effect of PTH. PTH-bound PTH1R engages with LRP6 and activates β-catenin in osteoblasts and osteocytes [23]. The activation of β-catenin by PTH is completely abrogated in *Lrp6-* deficient mice [18]. Similarly, the induction of total β-catenin levels that we observed in PTH-treated control mice was blunted in compound heterozygous animals. The related family member LRP5 cannot compensate for the inactivation or reduced expression of *Lrp6,* as the loss of *Lrp5* does not affect the anabolic actions of intermittent PTH [24].

Salt Inducible Kinases (SIKs) are critical transducers of the PTH signal as *Sik* gene deletion phenocopies PTH1R stimulation [25] and intermittent treatment with small molecule inhibitors of SIKs mimics iPTH administration [26]. The expression of SIK2 was strongly induced by iPTH treatment in compound *Naca/Lrp6* heterozygotes, but this enhanced expression could not compensate for the lack of iPTH responses in the mutant animals. A limitation of the interpretation of this experiment is that we assessed total SIK2 expression and not the PKA-induced phosphorylation of the protein. This was a technical limitation, as proteins precipitated from the phenol-ethanol supernatant of the TRIzol extracts from tibial shafts were not reactive to phospho-specific antibodies.

Gene-expression monitoring highlighted increased expression of the osteoblastic differentiation markers *Alpl* and *Col1a1* in iPTH-treated controls that was not present in compound heterozygotes. This suggests that the main effect of the PTH-PKA-NACA-LRP6 signal transduction pathway may be to stimulate osteoblast differentiation. The dual inactivation of one allele each of *Naca* and *Lrp6* reduced the expression of *Lrp6*, a transcriptional target of *Naca*. However, gene dosage manipulation of *Naca* did not affect its expression (data not shown). This was also observed in other models of compound heterozygosity involving *Naca* [27], suggesting that the control of the expression of *Naca* is not copy-number-dependent. However, every gene dosage manipulation involving *Naca* that we performed highlighted its genetic interaction as part of distinct signaling cascades [6,27].

One limitation of our study is that we did not perform histomorphometry or measure serum bone turnover markers to confirm that iPTH-stimulated bone formation is impacted in *Naca*^99A/A^ mutant mice and compound *Naca/Lrp6* heterozygotes.

The study design used in this project highlighted an important role of the PKA-NACA-LRP6 cascade for transduction of the iPTH signal. Obviously the numerous parallel pathways acting downstream from PTH that have been described previously (reviewed in [4,5]) are also involved in the physiological response to iPTH treatment. Their relative contributions could be exposed in different experimental setups with alternate strains, PTH doses or treatment regimen. Similarly, additional *Naca* transcriptional targets could be involved in the transmission of the PTH signal. The characterization of such additional *Naca* targets is ongoing in our laboratory [28].

## 4. Conclusions

Our results support a ‘feed-forward’ mechanism in which NACA plays a central role to prime subsequent iPTH responses by controlling *Lrp6* transcription to maintain a high amount of PTH1R/LRP6 complex at the plasma membrane.

## 5. Materials and Methods

### 5.1. Generation of Naca^99S/A^ Knock-in Strain

All animal procedures were reviewed and approved by the McGill Institutional Animal Care and Use Committee and followed the guidelines of the Canadian Council on Animal Care. Mice were kept in an environmentally controlled barrier animal facility with a 12-h light, 12-h dark cycle, and were fed mouse chow and water ad libitum.

*Naca*^99S/A^ knock-in mice were generated using a pMC-loxP-NEO-loxP vector (Specialty Media, Phillipsburg, NJ, USA) consisting of a loxP-NEO-loxP selection marker flanked by two fragments of the mouse *Naca* gene. A 3.7 kb fragment extending from exon 2 through intron 3 was cloned upstream of the selection cassette, and a 3.0 kb fragment covering from intron 3 through exon 9 was inserted downstream. Site-directed mutagenesis was used to mutate the serine residue at codon 99 (TCT) in exon 6 to alanine (GCT). The targeting vector was then linearized and electroporated into JM8.F embryonic stem (ES) cells [29]. Polymerase chain reaction (PCR) and Southern blot analysis were used to identify homologous recombination at the *Naca* locus. Positive clones identified as carrying the mutation were injected into C57BL/6 blastocysts. Male chimeric mice were mated with wild-type C57Bl/6 mice to produce heterozygous *Naca*^99S/A^ mice, and germline transmission was confirmed by DNA sequencing of mouse tail DNA. Mice were bred to CMV (cytomegalovirus)-Cre transgenic mice to excise the Neo selection cassette. Excision of the Neo selection cassette and segregation of the Cre transgene was confirmed using primers (5′-CTTTCTGGGAGTGGTTTGAAAGG-3′ and 5′-AGAGATGTGGGACAATAGCTAGAT-3′) flanking the Neo cassette. Heterozygous mice were interbred to generate wild-type (*Naca*^99S/S^), heterozygous (*Naca*^99S/A^), and homozygous mutant mice (*Naca*^99A/A^).

### 5.2. Generation of Compound Naca^99S/A^;Lrp6^+/fl^;Ocn-Cre Heterozygous Mice (Compound Heterozygotes)

The osteocalcin-Cre (OCN-Cre; B6.FVB-Tg(BGLAP-cre)^1Clem/J^) [22] and *Lrp6*-floxed mice [30] have been described previously. As a first breeding strategy to examine steady-state phenotypes, we crossed *Naca*^99S/A^ mice with OCN-Cre mice. From this cross, *Naca*^99S/A^;OCN-Cre progeny were bred to *Lrp6*^fl/fl^ mice. This allowed us to obtain all study genotypes in equivalent proportion: *Naca*^99S/S^; *Lrp6*^+/fl^; *Naca*^99S/S^; *Lrp6*^+/fl^; OCN-Cre; *Naca*^99S/A^; *Lrp6*^+/fl^; and *Naca*^99S/A^; *Lrp6*^+/fl^; OCN-Cre (compound heterozygotes).

To examine responses to iPTH treatment, *Naca*^99S/A^; OCN-Cre mice were crossed with *Lrp6*^+/fl^ mice to obtain test and control genotypes in equivalent proportion: (a) *Naca*^99S/S^; *Lrp6*^+/+^, (b) *Naca*^99S/S^; *Lrp6*^+/+^; OCN-Cre, (c) *Naca*^99S/S^; *Lrp6*^+/fl^, (d) *Naca*^99S/S^; *Lrp6*^+/fl^;OCN-Cre, (e) *Naca*^99S/A^;*Lrp6*^+/+^; OCN-Cre (f) *Naca*^99S/A^; *Lrp6*^+/+^, (g) *Naca*^99S/A^; *Lrp6*^+/fl^, and (h) *Naca*^99S/A^; *Lrp6*^+/fl^; OCN-Cre. After ascertaining that genotypes (a) to (e) had similar phenotypic manifestations (Supplementary Figure S5), we elected to compare genotype (b) (*Naca*^99S/S^; *Lrp6*^+/+^; OCN-Cre) as control and genotype (h) (*Naca*^99S/A^; *Lrp6*^+/fl^; OCN-Cre) as the test genotype (compound heterozygote). Genotypes (f) and (g), which were predicted to be phenotypically comparable to (a–e), were not analyzed.

### 5.3. Genotyping

All mice were genotyped using genomic DNA isolated from ear punches at 3 weeks of age. For the *Naca*^99S/A^ strain, the different genotypes were determined using a custom TaqMan single-nucleotide polymorphism (SNP) genotyping assay from Applied Biosystems (Life Technologies, Foster City, CA, USA) with *Naca* primers (forward: 5′-CTGGGTCTTCGACAGGTTACAG-3′ and reverse: 5′-CTCTTGTAGACATCGGGTTTTGTGA-3′) flanking the single-point mutation site. The 99S SNP primer (VIC-conjugated) was 5′-AGGATATTTTTAGATTTTCG-3′ and the 99A SNP primer (FAM-conjugated) was 5′-AGGATATTTTTAGCTTTTCG-3′ (mutated nucleotide underlined).

For the *Lrp6*-floxed strain, the genotyping used the following primers: forward 1 (F1): 5′- TGTGGGTAATGGACACGAGA-3′; forward 2 (F2): 5′- GTTGTCCACATTTGGGTTGA-3′; reverse 1 (R1): 5′-GAAACTAACCAGGCCCAAAG-3′. Primer pair F1/R1 yielded a wild-type amplimer of 230 bp and a floxed allele amplimer of 410 bp; primer pair F2/R1 generated a wild-type amplimer of 2 kb and a Cre-excised amplimer of 413 bp.

### 5.4. PTH Treatment

To determine the impact of *Naca* and *Lrp6* gene dosage manipulations on the anabolic response of bone to intermittent PTH (iPTH) treatment, 4-month-old compound heterozygous mice and control littermates were treated with 100 μg/kg of hPTH(1–34) (Cat. No. H4835, Bachem, Torrance, CA, USA) dissolved in PBS with 0.2 mM acetic acid or with vehicle (BSA, 1 mg/mL in PBS/0.2 mM acetic acid) subcutaneously, 5 times/week for 4 weeks. Mice were euthanized at the end of treatment for tissue collection.

### 5.5. Microcomputed Tomography (μCT)

Vertebrae (L3–L5) from each mouse were collected and cleaned of all surrounding soft and connective tissue, fixed overnight in 4% paraformaldehyde at 4 °C, and then washed with phosphate-buffered saline (PBS) (137 mM NaCl, 2.7 mM KCl, 10 mM Na2HPO4, 1.8 mM KH2PO4), dehydrated in 50% ethanol, then stored in 70% ethanol until ready for testing. The L4 vertebrae were analyzed using a SkyScan 1272 high-resolution μCT scanner (Bruker, Kontich, Belgium). Scans were acquired at an image pixel size of 4.5µm using a voltage of 52 kV, a current of 192 µA, a 0.25mm aluminum filter, an angular step of 0.4 degrees, an averaging frame of 3 and a 2 K resolution. The scans were reconstructed using the Skyscan NRecon Program (Bruker, Billerica, MA, USA). Reconstructions were performed using a ring artifact reduction value of 8, a smoothing value of 1 and a beam-hardening correction coefficient of 50–60%. The images were thresholded using two global threshold values, followed by a despeckling filter. We assessed the bone volume fraction (BV/TV, %), trabecular thickness (Tb.Th, mm), trabecular separation (Tb.Sp, mm), and trabecular number (Tb.N, mm^−1^).

### 5.6. Biomechanical Testing

The L2 vertebrae were dissected to remove the spinal cord and all tissue surrounding the vertebral body. The vertebrae were kept in PBS until testing to prevent drying. The vertebral crushing assay was performed using a Bose Electroforce 3200 Series III test instrument with a 225N load cell. Vertebrae were dried by dabbing onto a paper towel prior to insertion on the holder with the dorsal part within the compression area. Horns were oriented up for all samples. Samples were compressed at a rate of 0.1 mm/s. Data were collected in Wintest7 and analyzed using Matlab to derive stiffness (N/mm), load at yield (N), maximum load (N), and work to break (J). From the raw data, the percent change following PTH treatment was calculated and graphed.

### 5.7. Gene Expression Monitoring

Tibiae were dissected free of surrounding tissue and the epiphyses and metaphyses cut off prior to centrifugation to flush out the marrow. The diaphyses were then covered in RNA later (Ambion, Austin, TX, USA) and stored at −80 °C until ready for testing. Quantitative gene expression was assessed by real-time reverse transcriptase polymerase chain reaction (RT-qPCR). Briefly, tibial shafts were cut into small pieces. Bones were pulverized in 1 mL TRIzol reagent (Invitrogen, Carlsbad, CA, USA) using a benchtop tissue homogenizer (Brinkmann Kinematica Polytron PT 3000), and total RNA was isolated according to the manufacturer’s protocol. One (1) µg of RNA was reverse-transcribed to cDNA using a High Capacity cDNA Reverse Transcription Kit (Life Technologies Applied Biosystems, Carlsbad, CA, USA). Real-time qPCR using TaqMan universal PCR master mix and gene-specific Taqman assays for *Lrp6* (Mm00521783_m1), alkaline phosphatase (*Alpl*, Mm00475834_m1), and the alpha 1 chain of type I collagen (*Col1a1*, Mm00801666_g1) was performed using a QuantStudio 7 real-time PCR system (Life Technologies Applied Biosystems). The assay was performed in triplicate. Relative quantification of mRNA was performed according to the comparative C_t_ method and normalized to endogenous gene.

### 5.8. Immunoblotting

Following RNA isolation, proteins were precipitated from the phenol-ethanol supernatant of the TRIzol extract using isopropanol as per the manufacturer’s instructions. The pellet was resuspended in 1% SDS. For immunoblotting, an equal volume of 2X Laemmli loading buffer was added to 50 μg of cell extract. Proteins were denatured 5 min at 100 °C, resolved by SDS-PAGE on 7% acrylamide gels, transferred to PVDF membranes and immunodetected with anti-SIK2 (D28G3 rabbit monoclonal, Cell Signaling Technology catalog (Danvers, MA, USA) #6919; 1:1000), anti β-catenin (D10A8 rabbit monoclonal, Cell Signaling Technology catalog #8480; 1:1000) or anti-αTubulin (mouse monoclonal, Sigma-Aldrich catalog (St. Louis, MO, USA) #T6074; 1:1000) antibodies followed by incubation with appropriate secondary antibody. Blots were developed using the ECL Western blotting detection reagent according to the instructions of the manufacturer (GE Healthcare Bio-Sciences, Baie d’Urfé, QC, Canada).

### 5.9. Statistical Analysis

Statistical analysis was performed using GraphPad Prism version 7.04 (GraphPad Software Inc., San Diego, CA, USA) and one-way or two-way analysis of variance was used as appropriate, followed with the Bonferroni post hoc test. A probability value (*p*-value) lower than 0.05 was accepted as significant.

## Data Availability

Non applicable.

## Supplementary Materials

The following supporting information can be downloaded at: https://www.mdpi.com/article/10.3390/ijms23020940/s1.
