# Supplementary material for: NACA and LRP6 Are Part of a Common Genetic Pathway Necessary for Full Anabolic Response to Intermittent PTH
Source: Int J Mol Sci. 2022 Jan 15;23(2):940. doi: 10.3390/ijms23020940 (PMC8780913; doi:10.3390/ijms23020940)
Supplement: Supplementary file 1 [file ijms-23-00940-s001.zip › ijms-1530609-supplementary.pdf]

SUPPLEMENTARY FIGURES

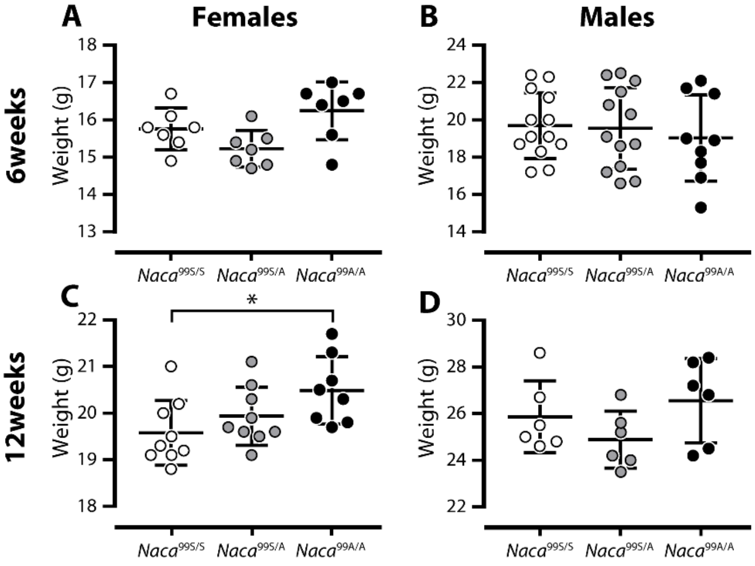

Supplementary Figure S1. Weight gain in *Naca*99S/A knock-in strain. Littermates of all genotypes and both sexes were weighted at 6 and 12 weeks of age. *, $p<0.05$, one-way analysis of variance (ANOVA) with post-hoc test.

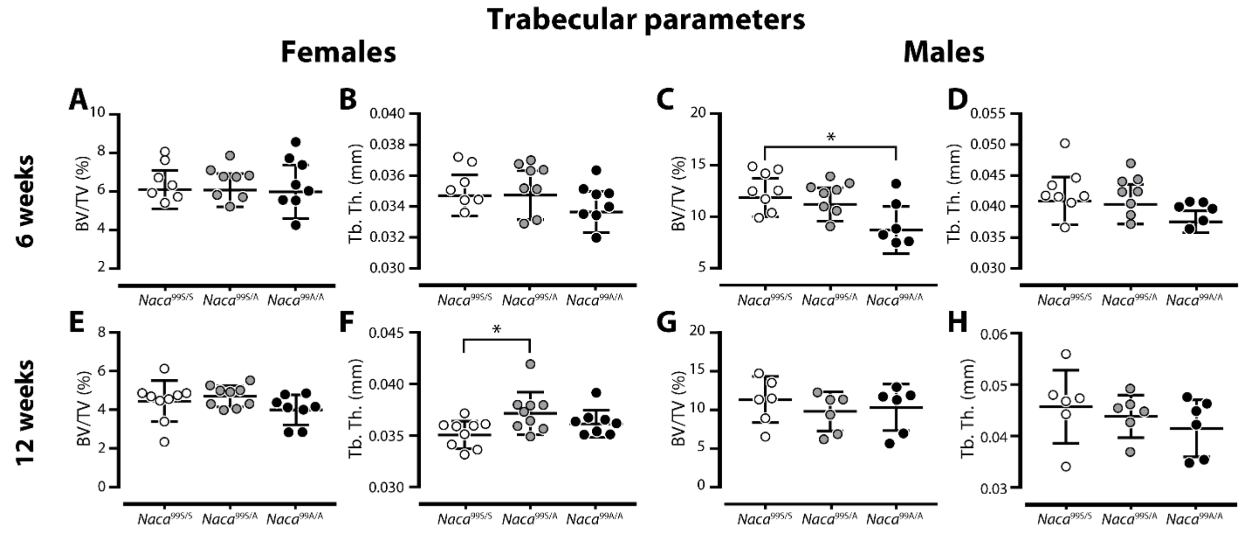

Supplementary Figure S2. Steady-state vertebral trabecular parameters in *Naca*$^{99S/A}$ knock-in strain. Vertebrae from littermates of all genotypes and both sexes were analyzed by mCT at 6 and 12 weeks of age. BV/TV, bone volume/tissue volume; Tb. Th., trabecular thickness. *, $p<0.05$, one-way analysis of variance (ANOVA) with post-hoc test.

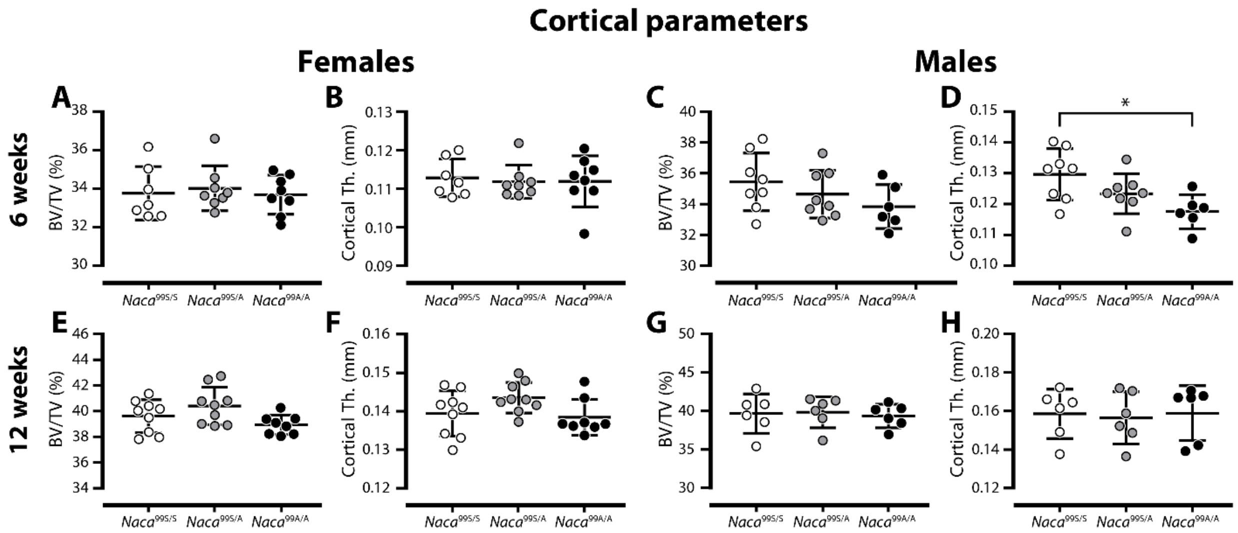

Supplementary Figure S3. Steady-state vertebral cortical parameters in *Naca*[99S/A] knock-in strain. Vertebrae from littermates of all genotypes and both sexes were analyzed by mCT at 6 and 12 weeks of age. BV/TV, bone volume/tissue volume; Cortical. Th., cortical thickness. *, $p<0.05$, one-way analysis of variance (ANOVA) with post-hoc test.

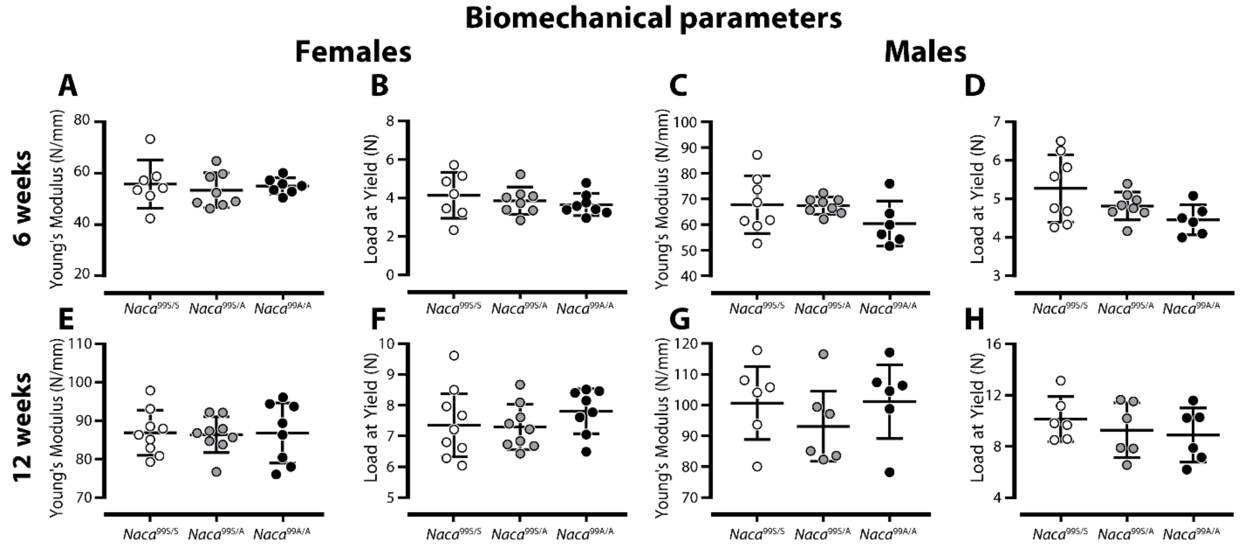

Supplementary Figure S4. Steady-state vertebral biomechanical parameters in *Naca*$^{99S/A}$ knock-in strain. Vertebrae from littermates of all genotypes and both sexes were analyzed by compression testing at 6 and 12 weeks of age.

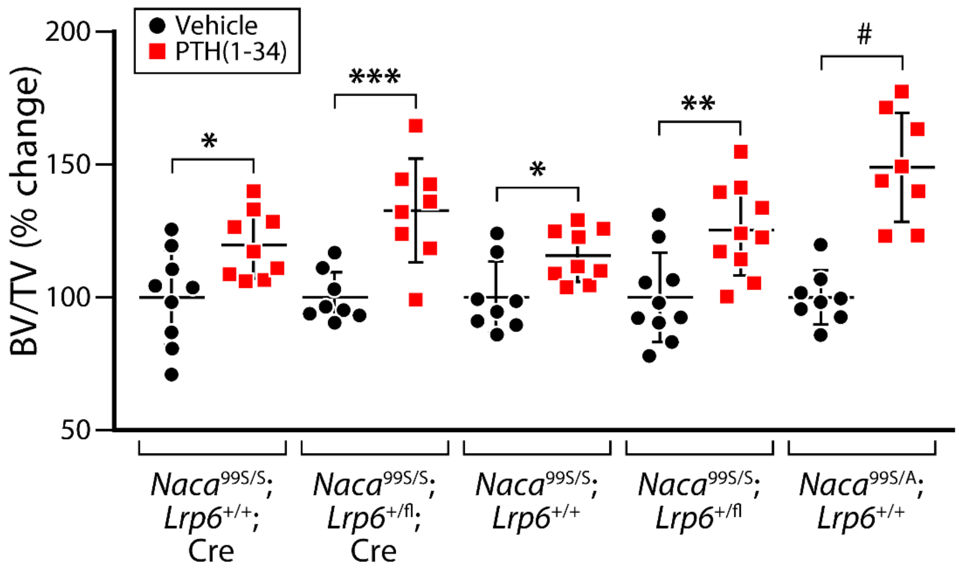

Supplementary Figure S5. Osteoanabolic response to iPTH in control genotypes. Four months-old female littermates of the indicated control genotypes were treated once daily, five days/week for one month with 100 mg/kg of PTH(1-34). Vertebrae were analyzed by mCT. BV/TV, bone volume/tissue volume. Results are expressed as % change from vehicle-treated animals. *, $p<0.05$; **, $p<0.01$; ***, $p<0.001$; #, $p<0.0001$, two-way ANOVA with post-hoc tests.
